# Bulked Segregant RNA Sequencing Revealed Difference Between Virulent and Avirulent Brown Planthoppers

**DOI:** 10.3389/fpls.2022.843227

**Published:** 2022-04-14

**Authors:** Wei Guan, Junhan Shan, Mingyang Gao, Jianping Guo, Di Wu, Qian Zhang, Jing Wang, Rongzhi Chen, Bo Du, Lili Zhu, Guangcun He

**Affiliations:** State Key Laboratory of Hybrid Rice, College of Life Sciences, Wuhan University, Wuhan, China

**Keywords:** brown planthopper, rice, insect virulence, biotype, bulked segregant RNA sequencing (BSR-seq)

## Abstract

The brown planthopper (*Nilaparvata lugens* Stål, BPH) is one of the most devastating insect pests of rice (*Oryza sativa* L.), but BPH populations have varying degrees of virulence to rice varieties carrying different resistance genes. To help efforts to characterize these variations we applied bulked segregant RNA sequencing (BSR-seq) to identify differentially expressed genes (DEGs) and genetic loci associated with BPH virulence to YHY15 rice plants carrying the resistance gene *Bph15.* BPHs that are highly virulent or avirulent to these plants were selected from an F2 population to form two contrasting bulks, and BSR-seq identified 751 DEGs between the bulks. Genes associated with carbohydrate, amino acid and nucleotide metabolism, the endocrine system, and signal transduction were upregulated in the avirulent insects when they fed on these plants. The results also indicated that shifts in lipid metabolism and digestive system pathways were crucial for the virulent BPHs’ adaptation to the resistant rice. We identified 24 single-nucleotide polymorphisms (SNPs) in 21 genes linked with BPH virulence. Possible roles of genes apparently linked to BPH virulence are discussed. Our results provide potentially valuable information for further studies of BPH virulence mechanisms and development of robust control strategies.

## Introduction

The brown planthopper (*Nilaparvata lugens* Stål, BPH) is a monophagous piercing-sucking herbivore that has become the most destructive insect pest of rice (*Oryza sativa* L.) (Sogawa, 1982; Hibino, 1996; Guo et al., 2018). BPHs occur widely throughout Asia and can migrate several 100 km across national borders (Jing et al., 2017). When feeding on rice plants, BPH nymphs and adults ingest phloem sap by inserting a stylet into their vascular tissues, thereby directly damaging the plants (Sogawa, 1982). They can also directly damage the plants by transmitting pathogenic viruses (Jena and Kim, 2010; Guo et al., 2018). Rice varieties that are resistant to BPH can inhibit the pest’s feeding, growth, and fecundity (Zheng et al., 2021). In recent decades, many BPH resistance genes and QTLs have been detected in cultivated rice and wild species (Guo et al., 2018; Shi et al., 2021; Zhou et al., 2021). However, new BPH biotypes may evolve that are virulent to resistant rice varieties (Claridge and Den Hollander, 1980; Saxena et al., 1991). A virulent biotype is defined as a BPH population that can damage a rice variety that was previously resistant (Claridge and Den Hollander, 1980; Jena and Kim, 2010). The first BPH-resistant variety (IR26 carrying the *Bph1* resistance gene) was developed by the International Rice Research Institute (IRRI) in 1973. However, within several years BPH biotype 2 had adapted and become virulent to IR26 in the field (Jena and Kim, 2010). Rice varieties resistant to biotype 2, carrying *Bph2*, were subsequently widely deployed in the field, but BPH biotype 3, which can overcome *Bph2*-mediated resistance, was detected in 1981 (Pathak and Heinrichs, 1982; Jena and Kim, 2010). Several additional BPH biotypes have been developed by rearing biotype 1 insects on specific resistant rice varieties in the laboratory (Claridge and Den Hollander, 1982). Analyses of various biotypes have clearly shown that BPH’s virulence to resistant rice varieties is a genetic trait (Kobayashi, 2016), but the mechanisms underlying BPH virulence are poorly understood.

Previous studies have shown that BPHs exhibit distinct responses to feeding on resistant plants carrying different resistance genes (Kobayashi, 2016; Yue et al., 2019). For instance, their responses to the resistant rice variety Mudgo, which carries the *Bph1* resistance gene, include activation of various metabolic, digestive, and absorption pathways (Ji et al., 2013). Wang et al. (2015) also found that feeding on the resistant rice variety B5 induced significant changes in expression of genes involved in metabolism of lipids, amino acids, carbohydrates, nucleotides, and TCA cycle intermediates in the salivary glands of BPH insects. Zhang et al. (2019) showed that expression levels of genes related to detoxification and starvation responses were significantly upregulated in BPHs that fed on resistant *Bph6*-transgenic rice. In addition, Zheng et al. (2020) observed stronger expression of genes involved in lipid metabolism in BPHs fed on NIL-*Bph6* and NIL-*Bph9* resistant rice plants than in insects fed on susceptible wild type rice. These studies have provided valuable insights. However, most data on BPH responses have been obtained by temporarily feeding avirulent insects on different rice varieties, and there have been few comparisons of virulent and avirulent BPHs fed on resistant varieties.

Various techniques have been used to study plant-insect interactions (Zogli et al., 2020). For instance, genome sequencing of the pea aphid showed that most chemosensory genes had undergone rapid expansion in the genome under positive selection (Smadja et al., 2009). Genome sequencing has detected the expansions of detoxification gene families in *Tetranychus urticae* (Grbić et al., 2011). The genome-wide analysis found the expansion of Cytochrome P450 (CYP) genes in the rice striped stem borer in response to rice defense secondary metabolites (Wang et al., 2014). MS/MS *de novo* peptide sequencing has found several enzymes responsible for digesting carbohydrates, proteins, and lipids in the midgut lumen of the cotton bollworm (Pauchet et al., 2008). In addition, insects have evolved effectors to adapt to host plants (Chen et al., 2019). The functional genomics approach has found effectors required for thrips to reproduce on tomatoes (Abd-El-Haliem et al., 2018). Additionally, some insect avirulence genes have been identified in Hessian fly associated with virulence to wheat R genes *H6* and *H13* by bulked segregant analysis (BSA)-seq analysis (Navarro-Escalante et al., 2020). A useful approach for studying disease resistance in plants is BSA, initially developed by Michelmore et al. (1991). BSA is based on the theory that different genotypes are likely to have differing phenotypic extremes, which can be used to rapidly identify genetic markers linked to genomic regions related to targeted traits (Michelmore et al., 1991; Wang et al., 2013). However, due to complications caused by repetitive genomic sequences and high genome sequencing depth requirements, BSA is not suitable for analyses of populations with numerous mutants and large genomes (Liu et al., 2012). Another approach, RNA sequencing (RNA-seq), is widely applied in next-generation sequencing (NGS) efforts to quantify relative quantities of specific transcripts from read counts (Górczak et al., 2020). RNA-seq reads can also be used to identify DNA sequence polymorphisms, such as single nucleotide polymorphisms (Chepelev et al., 2009). Bulked segregant RNA-seq (BSR-seq) couples BSA with RNA-seq (Liu et al., 2012) and can be used not only to identify differentially expressed genes (DEGs) but also single-nucleotide polymorphisms (SNPs) that are tightly linked to targeted traits (Du et al., 2017). BSR-seq has been successfully applied in analyses of many species. For instance, BSR-seq has identified 1,255 DEGs between catfish that are resistant and susceptible to enteric septicemia, and 56,419 SNPs of 4,304 unique genes of the fish (Wang et al., 2013). In addition, it has identified 18 high-probability SNPs and six genes that may be involved in responses to waterlogging stress in maize (Du et al., 2017), as well as the maize *glossy13* gene, which encodes a putative ABC transporter and is crucial for accumulation of epicuticular waxes (Li et al., 2013). As it combines advantages of BSA and RNA-seq it is highly efficient for analyses of complex traits involving multiple genes (Du et al., 2017; Li et al., 2020).

The BPH resistance gene *Bph15* was introduced into *O. sativa* from the wild rice species *Oryza officinalis* (Yang et al., 2004; Lv et al., 2014). Rice variety YHY15 is a recombinant inbred line (RIL) selected from the RI93 × TN1 F2 population and carries a single resistance gene *Bph15* (Yang et al., 2004). BPH population, designed biotype Y, was developed by rearing biotype 1 BPHs on YHY15 from January 2007 (Jing et al., 2012). Biotype Y can overcome the resistance conferred by *Bph15*, grow and reproduce normally on YHY15 plants. Thus, this combination of insects and plants provides a suitable model system for examining virulence mechanisms, and in this study we applied BSR-seq to identify DEGs and SNPs associated with BPH virulence to rice. Comparison of virulent and avirulent BPH extreme bulks after feeding on resistant YHY15 rice plants for 48 h identified 751 DEGs, with illuminating differences in transcriptomic profiles. Moreover, we identified 24 polymorphic SNPs related to BPH virulence located on six chromosomes, and 21 genes that may play key roles in virulence mechanisms. The results may facilitate further study of the mechanisms that enable BPH to overcome host resistance.

## Materials and Methods

### Insect and Plant Materials

The brown planthopper biotype 1 insects were reared on the susceptible rice variety Taichung Native 1 (TN1), which does not contain any BPH resistance gene. BPH biotype Y was developed by forcing biotype 1 BPHs to feed and reproduce on the resistant rice variety YHY15 for multiple generations (Jing et al., 2012). YHY15 is a RIL carrying the *Bph15* resistance gene (Yang et al., 2004). The BPHs used in the experiments reported here were reared on 1-month-old rice seedlings growing in 24 × 19 cm plastic buckets in controlled environmental conditions (26 ± 1°C, 16 h light/8 h dark cycles) at Wuhan University Institute of Genetics.

### Brown Planthopper Weight Gain and Honeydew Excretion Assay

The brown planthopper weight gain and honeydew excretion were measured as previously described (Shi et al., 2021). Newly emerged female adults were weighed using an AUW120D electronic balance (AUW120D, Shimadzu, Japan) then introduced into separate pre-weighed parafilm sachet (2 × 2.5 cm) fixed to leaf sheaths of a 4-week-old rice plant. After 48 h the insects were carefully removed from the sachets, and then the insect and the honeydew in each sachet were weighed. Each insect’s weight gain was determined from its weight before and after ingestion, and its weight gain ratio was calculated by dividing its weight gain by its initial weight. Both BPH weight gain and honeydew excretion assays were repeated three times with 25 BPHs for each replicate.

### Brown Planthopper Survival Rate

The brown planthopper survival rates were determined as previously described (Shi et al., 2021). Briefly, rice plants were grown in plastic pots (12 cm tall, 9 cm diameter, one plant per pot). When they were 1-month-old the pots were covered with plastic cages and 10 third-instar nymphs were released into each cage to infest the plants. The number of surviving BPH nymphs in each cage was recorded every day until 10 days after their introduction, and survival rates were calculated by dividing the number of surviving nymphs in each cage by the number initially released. The experiment was repeated three times, with 20 BPHs per replicate.

### Formation of Brown Planthopper Extreme Bulks

An extremely virulent BPH female biotype Y adult, which gained 2.06 mg weight in the weight gain assay described above, was mated with an avirulent male adult of biotype 1. The F1 offspring were allowed to intermate and female F2 adults resulting from their mating, with similar body sizes, were subjected to the assay. Forty that gained 1.89–2.8 mg weight were classified as virulent to YHY15, and 40 that lost 0.4–1.04 mg were classified as avirulent. These sets were used to form a *Bph15*-virulent bulk (vB15) and a *Bph15*-avirulent bulk (aB15), respectively, which were used to prepare bulked RNA samples for the BSR-seq analysis.

### Illumina cDNA Library Construction and RNA Sequencing

Each BPH individual in the extreme bulks was dissected into two parts under an Olympus stereoscopic microscope, the heads with intestines and the remaining carcasses. The two resulting bulks from the sets of 40 virulent and avirulent F2 individuals were each randomly divided into four replicates, with parts of 10 insects in each replicate. The tissue samples in each replicate were mixed, and total RNA was extracted from them using a mirVana miRNA Isolation Kit (Ambion-1561, Austin, TX, United States). After testing its integrity using a 2100 Bioanalyzer (Agilent Technologies, Santa Clara, CA, United States), sequencing libraries were constructed using a TruSeq Stranded mRNA LTSample Prep Kit (Illumina, San Diego, CA, United States) following the manufacturer’s instructions. The cDNA library preparations were then sequenced using an Illumina HiSeq 4000 sequencing platform. Raw reads were processed using Trimmomatic (Pérez-Rubio et al., 2019). To obtain clean data, reads containing poly-n and low-quality reads were removed. Then the clean reads were used for the BSR-seq analysis.

### SNP Calling and Bulked Segregant RNA-Seq Analysis

The reads were mapped to the latest chromosome-level BPH reference genome (BioProject accession no. PRJNA591478) (Ma et al., 2021) using Burrows–Wheeler Aligner (BWA, v. 0.7.5a) (Rajan-Babu et al., 2021). The bwa index command and BWA–MEM were used to create reference genome index and map the reference genome, respectively. Then, the mapped reads were sorted and indexed by SAMtools (v. 1.3.1) (Danecek et al., 2021), following recommended procedures.^1^ SNPs were called using SAMtools and BCFtools (v. 1.3.1) (Lam et al., 2020). The Variant Call Format (VCF) files are shown in Supplementary Materials. Then SnpEff v. 4.1g (Cingolani et al., 2012) was used to annotate and predict the effects of genetic variants on genes. And SNP index was calculated at each SNP position to indicate the proportion of reads harboring SNPs that differed from the reference sequences (Abe et al., 2012). SNP positions with SNP index less than 0.3 were eliminated, and those with depth less than four were excluded, as these SNPs may be due to sequencing or alignment errors (Hisano et al., 2017; Feng et al., 2020; Li et al., 2020). After filtration, ΔSNP-index value (Takagi et al., 2013; Li et al., 2020) was calculated for each position by subtracting the SNP index of the aB15-bulk from that of the vB15-bulk. The average ΔSNP-index was calculated using a sliding window analysis with a window size of 3-Mb and a slide size of 1-Mb. We computed confidence intervals of ΔSNP-index values for all SNP positions under the null hypothesis that no QTLs were present.

### Differential Expression Gene Analysis

The clean reads were mapped to the BPH reference genome (Ma et al., 2021) using Hisat2 (v. 2.2.1.0) software^2^ (Kim et al., 2015). To quantify gene expression levels, fragments per kilobase of transcript per million mapped reads (FPKM) values (Roberts et al., 2011) were calculated using Cufflinks (Trapnell et al., 2010), and read counts of each gene were determined by htseq-count (Srinivasan et al., 2020). DEGs were clustered using the DESeq package (Anders and Huber, 2012) with thresholds of log_2_FC ≥ 1 and *P* < 0.05 for significantly differential expression. The DEGs’ expression patterns were explored by hierarchical cluster analysis and they were functionally annotated by comparing with entries in the Nr, eukaryotic orthologous groups (KOG), Swiss-Prot, Gene ontology (GO), Pfam, and Kyoto Encyclopedia of Genes and Genomics (KEGG) databases (with *P* ≤ 0.05 as the threshold in GO term and KEGG pathway enrichment analyses).

### Real-Time Quantitative PCR Analysis

For expression analyses of candidate genes in *Bph15*-virulent (Vr-B15) and *Bph15*-avirulent (Avr-B15) BPHs. The weight gain of newly emerged female adults from biotype Y was measured after feeding on YHY15 for 48 h. The virulent insects (average weight gain in 2.0 mg) and the avirulent (weight gain –0.4 mg) were used for qRT-PCR, respectively. Total RNA was isolated from the insects using a RNAiso Plus kit (Takara, Kyoto, Japan) following the manufacturer’s instructions. RNA samples were reverse-transcribed into cDNA using a PrimeScript RT Reagent Kit with gDNA Eraser (Takara, Kyoto, Japan). The resulting sequences were then used in qRT-PCR analysis with a CFX96 Touch™ Real-Time System and iTaq Universal SYBR Green Supermix Kit (Bio-Rad, Hercules, CA, United States) according to the manufacturer’s protocols. Relative expression levels were calculated using the 2^–ΔΔCt^ method with the BPH housekeeping gene *actin* serving as an internal control. Three independent biological replicates were employed in each experiment.

### Statistical Analysis

The significance of differences in recorded variables was assessed using Student’s *t*-test or Tukey’s honestly Significant Difference test implemented in IBM SPSS Statistics v. 23.0. Differences were considered significant, very significant and highly significant (indicated by *, ^**^, ^***^ in the figures) if *P* < 0.05, *P* < 0.01, and *P* < 0.001, respectively.

## Results

### The Performance of Brown Planthopper Insects on YHY15 Rice Plants

**FIGURE 1:**
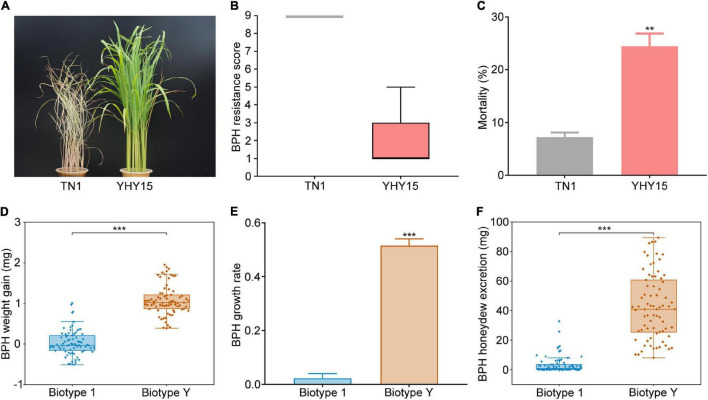
Bph15-mediated resistance verification and virulence assessment of BPH biotype Y. **(A)** Photo showing the difference in TN1 and YHY15 rice plants’ BPH resistance. **(B)** BPH-resistance scores of TN1 and YHY15 plants (negatively related to BPH resistance) obtained from observations 7 days after BPH infestation (*n* = 3 independent experiments, each with 15 rice plants). **(C)** Mortality of BPH insects fed on TN1 and YHY15 plants after 48 h. Weight gain **(D)** and growth rate **(E)** of BPH biotypes 1 and biotype Y feeding on YHY15 rice plants for 48 h. Three independent experiments were repeated with 25 BPHs per replicate. **(F)** Amounts of honeydew excreted by newly emerged female adults of biotype 1 and biotype Y on YHY15 plants after 48 h. Three independent experiments were performed with 25 BPHs per replicate. All data presented are means ± SE of three replicates. In the box charts, the bottom and top edges of the box charts represent the 25th and 75th percentiles, respectively, and center values are medians. The whiskers mark ranges of the data, excluding outliers. **, *** on the bars indicate significance at *P* < 0.01, and *P* < 0.001 (according to Student’s *t*-test), respectively.

To verify the BPH resistance conferred by *Bph15*, YHY15 plants carrying *Bph15* were used for seedling stage insect resistance analysis, with susceptible TN1 plants as controls. After infestation by biotype 1 BPHs for 7 days, the TN1 plants were dead, but the YHY15 plants survived well, proving YHY15’s resistance to biotype 1 BPHs (Figures 1A,B and Supplementary Figure 1). Moreover, the mortality rate of biotype 1 insects was also significantly higher on YHY15 (24.44%) than on TN1 (7.22%) plants (Figure 1C). These results clearly indicate that the YHY15 plants were resistant to BPH.

To further investigate the virulence of BPH biotype Y to YHY15 rice, we observed the performance of adult female biotype 1 and Y insects (with similar initial body weight, ranging from 1.8 to 2.7 mg) on YHY15 plants. After feeding on YHY15 plants for 48 h, the biotype Y and 1 insects had average weight gain of 1.08 and 0.03 mg, respectively (Figure 1D), with average growth rate of 51.58 and 2.24%, respectively (Figure 1E). In addition, the biotype Y and 1 BPHs had average honeydew excretion of 42.78 and 3.55 mg, respectively (Figure 1F). These results indicate that the biotype Y has adapted to the resistant YHY15 rice plants.

### Construction of Extremely Virulent and Avirulent Brown Planthopper Bulks

**FIGURE 2:**
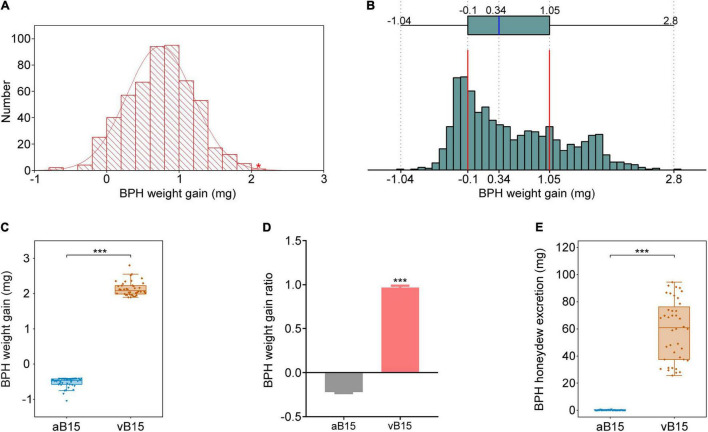
Construction of the two extreme BPH bulks. **(A)** Frequency distribution of weight gain of 504 biotype Y BPHs after feeding on YHY15 plants for 48 h. A single extremely virulent BPH female, with 2.06 mg weight gain (indicated by the red asterisk), was selected. **(B)** Frequency distribution of weight gain of F2 BPHs (*n* = 1202) fed on YHY15 plants for 48 h. The histogram shows the BPHs’ weight gain. The chart above it shows the range of BPH weight gain, with 25th and 75th percentiles (excluding outliers) indicated by right and left edges of the box, respectively, and the median within the box. Panels **(C–E)** show the weight gain, weight gain ratio, and honeydew excretion, of the two bulks after feeding on YHY15 for 48 h (three independent experiments were performed). vB15-bulk and aB15-bulk refer to BPHs extremely virulent and extremely avirulent to YHY15, respectively. ***P < 0.001 (Student’s *t*-test).

To construct the virulent and avirulent BPH bulks, 504 BPH female adults of biotype Y were randomly selected and we recorded the weight gain of each insect after feeding on YNY15 plants for 48 h. Their weight gain of BPH insects was continuously distributed in range of –0.79–2.06 mg (Figure 2A), indicating significant heterogeneity in virulence in the biotype Y population. Therefore, a virulent female with 2.06 mg weight gain from biotype Y (indicated by the red asterisk in Figure 2A) was selected and crossed with an avirulent male of the biotype 1 population. The emerging male and female offspring were then intercrossed to breed a F2 generation for bulk construction.

In total, the virulence of 1202 newly emerged F2 female adults was assessed by allowing them to feed on resistant YHY15 plants for 48 h and recording their weight gain, which ranged from –1.04 to 2.8 mg (Figure 2B), clearly showing that their virulence widely varied. We selected 40 extremely virulent and 40 avirulent BPHs with weight gains ranging from 1.89 to 2.8 mg and weight losses ranging from 0.4 to 1.04 mg, respectively (Figure 2C), and average weight gain ratios of 96.49 and –22.27%, respectively (Figure 2D). In addition, BPH honeydew excretion is a reliable indicator of BPH feeding activity. It has been applied to determine the virulence of the biotypes (Claridge and Den Hollander, 1980). The virulent BPHs produced 25.62–94.45 mg honeydew, and the avirulent 0–0.77 mg honeydew, respectively (Figure 2E). Hence, the 40 extremely virulent F2 insects and 40 extremely avirulent counterparts were designated the *Bph15*-virulent (vB15) bulk and *Bph15*-avirulent (aB15) bulk, respectively (Supplementary Figure 2).

### Transcriptome Annotation of the Assembled Unigenes

We subjected the two extreme BPH bulks to deep RNA-sequencing. For this, the 40 F2 individuals in each extreme bulk were randomly divided into four replicates with 10 insects in each replicate, and each insect was divided into two parts (heads + intestines and remaining carcasses). RNA transcripts were extracted from the pooled pairs of tissue samples of each set of 10 BPHs then sequenced using an Illumina HiSeq 4000 sequencing platform, generating eight transcriptome libraries with 59.6 G raw data in total. After filtering out low-quality sequences, incorrect adapters, and redundant sequences, 55.67 G clean data were obtained. In addition, 82.09–86.03% of clean reads were mapped to the latest chromosomal-level BPH reference genome (Ma et al., 2021), and the Q30 percentage exceeded 91.58% (Table 1).

**TABLE 1:** Summary of the RNA sequencing data.

Sample	Raw bases	Clean bases	Q30 (%)	GC (%)	Total mapped reads	Mapping ratio (%)
aB15_1	7.14G	6.61G	91.94	42.33	38742958	85.87
aB15_2	7.38G	6.83G	91.89	42.42	40139950	86.03
aB15_3	7.41G	6.84G	91.58	42.67	40055545	85.69
aB15_4	7.76G	7.20G	92.13	42.16	40356826	82.09
vB15_1	7.35G	6.81G	92.08	41.37	38729806	83.34
vB15_2	7.46G	7.04G	93.51	42.16	39970460	83.47
vB15_3	7.38G	7.01G	93.91	40.40	39937855	83.91
vB15_4	7.72G	7.33G	94.05	40.97	41755631	83.82
Total	59.6G	55.67G	–	–	–	–

**FIGURE 3:**
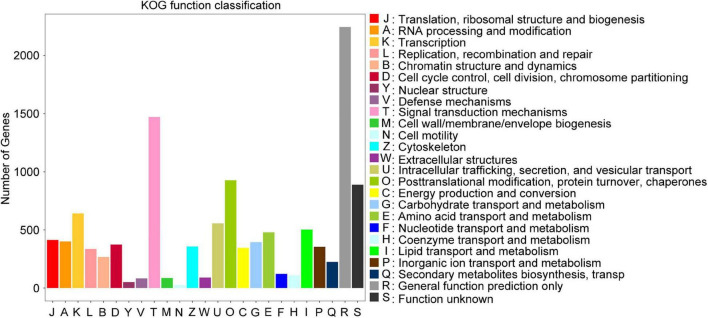
Histogram of numbers of genes in orthologous group classes. In total 10,450 unigenes were annotated into 25 KOG categories and the number of unigenes in each specific functional clump is indicated on the *y*-axis.

Results of the correlation analysis showed that the gene expression profiles of the four biological replicates of each bulked RNA sample correlated well (Supplementary Figure 3 and Supplementary Table 1). Principle component analysis (PCA) showed that the first two principal components (PC1 and 2) explained 61 and 12.7% of the variance, respectively. Samples within each group clustered together (Supplementary Figure 4). In particular, 18,559 unigenes were identified based on the clean reads obtained from the two BPH extreme bulks. Based on the sequence homology, 10,450 unigenes were assigned to KOG functional classifications. These included 25 KOG categories, most commonly general function prediction only (21.48%), followed by: signal transduction mechanisms (14.08%); post-translational modification, protein turnover, chaperones (8.871%); transcription (6.134%); intracellular trafficking, secretion, and vesicular transport (5.33%); lipid transport and metabolism (4.813%); amino acid transport and metabolism (4.584%); and translation, ribosomal structure and biogenesis (3.962%) (Figure 3). Transcripts of numerous genes involved in basic processes such as translation and transcription, as well as functions including lipid transport, signal transduction, and post-translational modification mechanisms were detected.

### Transcriptomic Profiles of Avirulent and Virulent Brown Planthoppers

**FIGURE 4:**
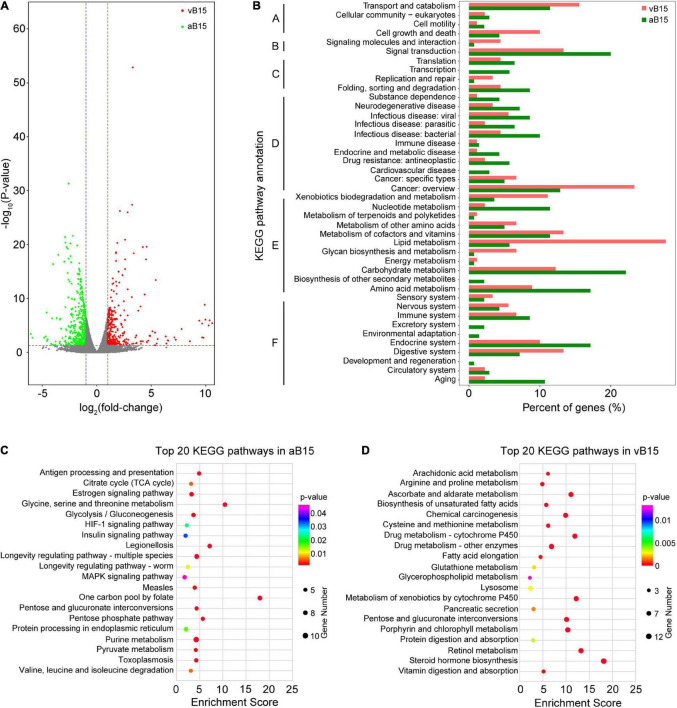
Functional analyses of the differentially expressed genes (DEGs). **(A)** Volcano plots of DEGs (*P* < 0.05 and log2FC = 1.) between the vB15- and aB15-bulks, based on log2FC and FDR values. Green and red scatterpoints indicate DEGs more strongly expressed in the aB15-bulk and vB15-bulk, respectively. The results are based on comparison of expression of genes in vB15-bulk and aB15-bulk. **(B)** Kyoto Encyclopedia of Genes and Genomes (KEGG) classification of the DEGs more strongly expressed in the aB15- and vB15-bulks, respectively. The DEGs were assigned to KEGG pathways associated with: (A) Cellular processes; (B) Environmental information processing; (C) Genetic information processing; (D) Human diseases; (E) Metabolism; (F) Organismal systems. The DEGs more strongly expressed in the aB15-bulk were assigned to 42 KEGG categories, and those more strongly expressed in the vB15-bulk were assigned to 36 categories. **(C,D)** The 20 most highly enriched KEGG pathways in the aB15 and vB15 bulks, respectively.

Transcriptomic profiles of the vB15- and aB15-bulks were compared to identify DEGs with fold-change (FC) ≥ 2 and *P* < 0.05 (Supplementary Figure 5). In total, 751 DEGs were detected (Supplementary Table 2), of which 418 were more strongly expressed in the aB15-bulk and 333 in the vB15-bulk (Figure 4A). These findings indicate that feeding on the YHY15 plants induced stronger transcriptomic changes in the avirulent BPHs than in the virulent BPHs. We subsequently subjected to the DEGs to gene ontology (GO) analysis to identify the key processes that were enriched in each extreme bulk. The GO analysis (level 2) illustrated that GO terms that were mainly enriched in both aB15 and vB15-bulks were involved in single-organism, cellular, metabolic, binding, and catalytic activity processes (Supplementary Figure 6 and Supplementary Table 3). Additionally, KEGG analysis (level 2) revealed that the 418 DEGs more strongly expressed in the aB15-bulk were mainly involved in: carbohydrate, amino acid and nucleotide metabolism; signal transduction; the endocrine system; and protein folding, sorting and degradation (Figure 4B). These changes indicate that *Bph15*-mediated resistance mechanisms induced substantial changes in diverse physiological processes of avirulent BPHs. The 333 DEGs more strongly expressed in the vB15-bulk were mainly assigned to lipid metabolism and digestive system categories (Figure 4B), suggesting that these pathways are crucial for BPH adaption to *Bph15*-mediated resistance.

We also subjected the DEGs to KEGG enrichment analysis (with a *P* < 0.05 threshold) to further pinpoint key pathways in each bulk. The 418 more strongly expressed in the aB15-bulk were mainly involved in: purine, carbohydrate, and amino acid metabolism; longevity regulation; and MAPK signaling pathways. Genes associated with six amino acids (Gly, Ser, Thr, Val, Leu, and Ile) were more strongly expressed in the aB15-bulk after feeding on resistant YHY15 rice plants. Genes of the glycolysis/gluconeogenesis, citrate cycle (TCA cycle), and pentose phosphate pathways of carbohydrate metabolism categories were significantly enriched in the avirulent BPHs (Figure 4C and Supplementary Table 4). In contrast, assignations of the 333 DEGs more strongly expressed in the vB15-bulk indicated significant enrichment of lipid metabolism (including steroid hormone and unsaturated fatty acid biosynthesis, fatty acid elongation, arachidonic acid metabolism, and glycerophospholipid metabolism pathways) in the virulent insects. Pancreatic secretion, vitamin digestion and absorption, and protein digestion and absorption pathways of the digestive system category were also significantly enriched in the vB15-bulk (Figure 4D and Supplementary Table 4). These results indicate that the virulent BPHs’ adaption to YHY15 is related to lipid metabolism, protein digestion, and absorption pathways.

### Verification of Differentially Expressed Genes in Key Pathways

**FIGURE 5:**
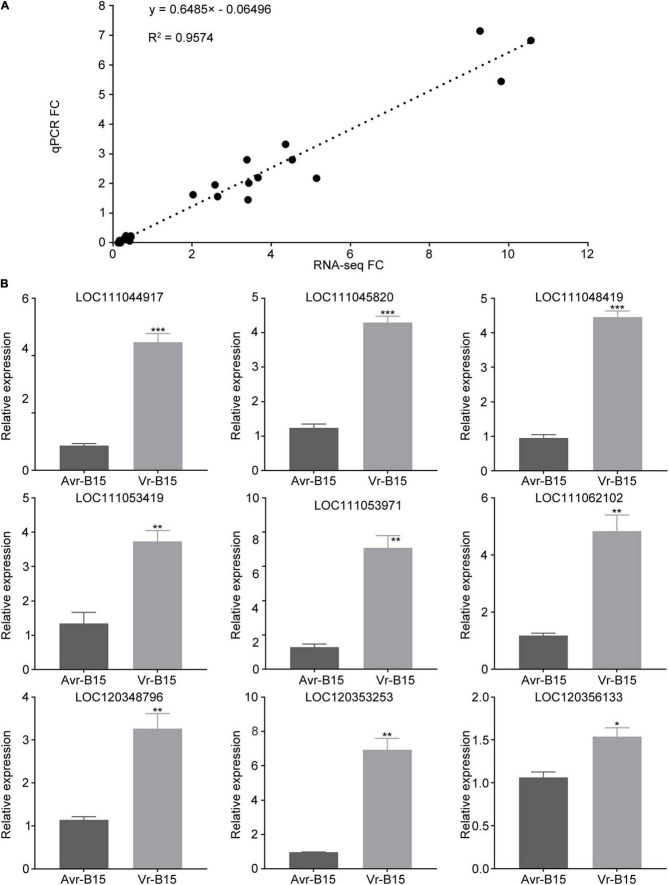
Validation of the transcriptome sequencing quality. **(A)** Correlation of the RNA-seq and RT-qPCR results. Expression levels of 30 randomly selected DEGs, including 13 and 17 more strongly expressed in the vB15-bulk and aB15-bulks, according to analyses of the RNA samples (normalized with respect to the BPH β*-actin* gene; three biological replicates). Fold-change values of these genes obtained from the RNA-seq and RT-qPCR analyses are strongly positively correlated. **(B)** Expression levels of nine DEGs related to lipid metabolism in Avr-B15 and Vr-B15 (avirulent and virulent BPHs with 0.4 mg weight loss and 2.0 mg weight gain after feeding on a YHY15 plant for 48 h, respectively). Presented data are means ± SE of three replicates. Error bars indicate SEM, *n* = 3 independent experiments. Asterisks above the bars indicate significant differences, according to Student’s *t*-test (*P < 0.05; **P < 0.01; ***P < 0.001).

To verify the reliability of the RNA sequencing data, we validated 30 randomly selected DEGs by quantitative reverse transcription-PCR (qRT-PCR) analysis with gene-specific primers (Supplementary Table 5). Expression levels of these 30 randomly selected DEGs (13 and 17 that were more strongly expressed in the vB15-bulk and aB15-bulk, respectively) were determined in both bulks, normalized with respect to *actin* expression, and then compared with the corresponding transcript read counts. Their expression levels according to the RNA-seq and qRT-PCR analyses correlated extremely well, with an *R*^2^ value of 0.9574 (Figure 5A), clearly confirming the credibility of our RNA-seq data.

We also analyzed nine DEGs in the lipid metabolism pathway that were significantly enriched using qRT-PCR (Supplementary Table 5). These nine DEGs’ annotations indicate that they are UDP-glucuronosyltransferases (Supplementary Table 2), which play diverse roles, including detoxification (Bock, 2016), by catalyzing the transfer of sugar molecules to lipid and protein acceptors (Zielinski et al., 2016). Expression levels of these genes were examined, by qRT-PCR, in representatives of the *Bph15*-virulent (Vr-B15) and *Bph15*-avirulent (Avr-B15) insects of biotype Y, with 2.0 mg weight gain and 0.4 mg weight loss after feeding on YHY15 plants for 48 h, respectively. The results showed that all these genes were expressed significantly more strongly in the Vr-B15 insect than the Avr-B15 insect (Figure 5B). These results are consistent with the pathway enrichment results, indicating that lipid metabolism pathways were regulated differentially in the virulent and avirulent insects in response to rice plants carrying the *Bph15* resistance gene.

### Identification of Single-Nucleotide Polymorphisms Linked to Virulence

**FIGURE 6:**
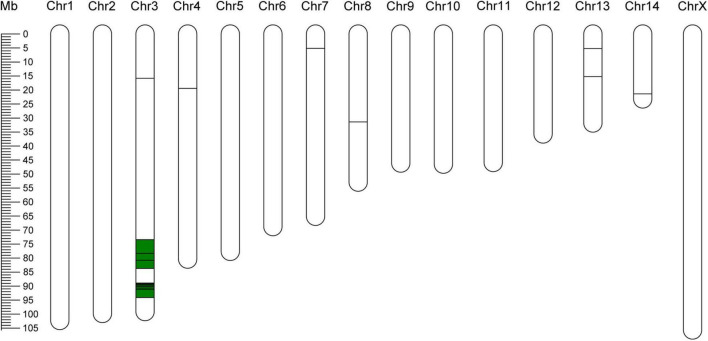
Distribution of polymorphic SNPs on chromosomes. The horizontal lines indicate positions of SNPs with a significance level of 99%. The green blocks indicate chromosome regions linked to BPH virulence.

In total, 63127 SNPs with high confidence were obtained from the transcriptomic data of the two bulks. Application of the ΔSNP-index method identified 24 SNPs localized in 21 genes on six chromosomes at a significance level of 99% (Supplementary Table 6; Feng et al., 2020; Li et al., 2020; He et al., 2022). Moreover, two chromosome regions harboring SNPs were identified on chromosome 3 (73413752-83702207 and 88874910-94058321), as shown in Figure 6 and Supplementary Figure 7. In addition, 33 of the 751 DEGs were located near identified SNPs linked to virulence (Supplementary Table 7). These results clearly suggest that multiple loci control BPH virulence to plants with *Bph15*-mediated resistance.

Putative functions of the 21 genes hosting the 24 identified SNPs were annotated (Supplementary Table 8). One of these genes, *LOC111057305*, encodes a fatty acyl-CoA reductase (FAR), which plays a crucial role in insect pheromone biosynthesis (Teerawanichpan et al., 2010). Another, *LOC111052455*, encodes a member of the aldo–keto reductase 6 (AKR6) family involved in channel trafficking and membrane localization (Barski et al., 2009). One of these genes, *LOC111054236*, encodes a dipeptidyl peptidase 4 (DPP 4) protein, a vital digestive peptidase in *Tenebrio molitor* larvae (Tereshchenkova et al., 2016). Another, *LOC111046750*, encodes a peroxidasin homolog protein involved in basement membrane biogenesis, tissue development, and innate immune defense (Nelson et al., 1994). And *LOC120354300* encodes a zinc finger protein, which has diverse functions, including lipid binding, transcriptional activation, regulation of apoptosis, and protein folding and assembly (Laity et al., 2001). Others include *LOC111055290*, an integrator complex subunit implicated in adipose differentiation (Otani et al., 2013), and *LOC111048257*, encoding a basement membrane specific heparan sulfate proteoglycan core protein. These candidate genes encode enzymes, zinc finger proteins, and other functional proteins that may participate in BPH virulence to plants with *Bph15*-mediated resistance.

## Discussion

Previous studies have revealed significant genetic variations in virulence within BPH populations (Den Hollander and Pathak, 1981; Tanaka, 1999). Accordingly, this study found substantial variation in the virulence of randomly selected individuals of BPH biotype Y (Figure 2A). Thus, direct comparison of wild BPH populations may not readily identify genetic factors of BPH virulence (Via, 1990; Kobayashi, 2016). The heterogeneity of the parental BPH population also reportedly complicates inheritance of BPH virulence (Sogawa, 1981). Therefore, we mated a virulent female and an avirulent male to produce a F1 generation, and F1s were intermated to produce the F2 generation. Two BPH extreme bulks were constructed from the F2 population by the BSR approach. Virulence assessment validated these two extreme bulks (Figure 2), then 751 DEGs and 24 SNPs in 21 candidate genes were identified by BSR-seq analysis, confirming its suitability for studying BPH biotype Y’s virulence to rice plants with *Bph15*-mediated resistance. To our knowledge, this is the first reported use of the BSR-seq method to identify candidate genes involved in BPH virulence to rice resistance.

Functional analyses of the identified DEGs showed that feeding on plants with *Bph15*-mediated resistance induced more extensive transcriptomic changes in the avirulent BPHs than in the virulent insects. Carbohydrates are the main chemical components in the phloem sap of rice and essential nutrients for phloem-sucking insects (Foyer et al., 2007). In this study, we detected 42 DEGs associated with carbohydrate metabolism in the two BPH bulks. More of these were expressed more strongly in avirulent BPHs than in virulent BPHs (Figure 4B). The expression patterns also showed that the glycolysis/gluconeogenesis, citrate cycle (TCA cycle), and pentose phosphate pathways were significantly enriched in the avirulent BPHs after feeding on resistant YHY15 rice plants (Figure 4B). These findings indicate that feeding on YHY15 plants induces upregulation of carbohydrate metabolism in avirulent BPH. We also found that it induced significant increases in expression of genes involved in synthesis of six amino acids (Gly, Ser, Thr, Val, Leu, and Ile) in the avirulent BPHs (Figure 4C). This has clear physiological implications as amino acids are the raw materials for protein synthesis and participate in myriads of fundamental processes in insects, including energy provision (Hargrove, 1976), sclerotization of newly formed cuticle (Andersen, 2007), and detoxification of harmful exogenous substances (Novoselov et al., 2015). Similarly, Peng et al. (2017) found that levels of six amino acids (Val, Leu, Ser, Thr, Pro, and Gln) were higher in BPHs fed on resistant YHY15 rice plants than in counterparts fed on susceptible TN1 rice. Moreover, Liu et al. (2017) found that levels of 19 amino acids were lower in BPH nymphs after feeding on YHY15 rice than after feeding on TN1 plants for 12 h. These results suggest that the higher amino acid levels may contribute to the feeding and survival of avirulent BPHs on resistant YHY15 rice. MAPK signaling, purine metabolism, and longevity regulating pathways were also enriched in the avirulent BPHs after feeding on the resistant rice (Figure 4C). Collectively, these findings suggest that feeding on rice with *Bph15*-mediated resistance may perturb diverse physiological processes in avirulent BPHs.

In contrast, feeding on the YHY15 plants more specifically altered lipid metabolism in virulent biotype Y BPHs, in accordance with previous findings that lipid mobilization in their bodies is crucial for BPHs feeding on resistant rice plants (Yue et al., 2019). In this study, we found that it induced stronger expression of lipid metabolism-related pathways in virulent BPHs than in avirulent BPHs. Biosynthesis of steroid hormones and unsaturated fatty acids, fatty acid elongation, arachidonic acid metabolism, and glycerophospholipid metabolism pathways were significantly enriched in the virulent-BPH bulk (Figure 4D). These results indicate that lipid metabolism pathways are important for BPH virulence to *Bph15* resistance, and are consistent with previous reports that lipid metabolism in BPH fat bodies is activated when the insects feed on rice plants carrying *Bph6* or *Bph9* resistance genes (Zheng et al., 2020). In addition, efficient digestion and nutrient uptake are vital for insects to acquire essential nutrients from their diet (Holtof et al., 2019). Comparative analysis of salivary gland transcriptomes of two BPH populations derived from TN1 and Mudgo has revealed that genes related to “digestion and absorption” might be associated with BPH virulence (Ji et al., 2013). Results presented here indicate that feeding on the resistant plants induced stronger expression of pathways involved in the digestive system, including pancreatic secretion, as well as the digestion and absorption of vitamins and proteins in virulent BPHs (Figures 4B,D). These findings corroborate the importance of the digestive system’s importance in BPH virulence. In sum, the findings indicate that lipid metabolism and digestive system pathways are strongly involved in BPHs’ adaptation to resistant YHY15 plants.

Previous studies showed that the polygenic system controls BPH virulence (Claridge and Den Hollander, 1980; Tanaka, 1999; Jing et al., 2017). For example, BPH virulence to rice plants with *Bph1*-mediated resistance is influenced by a recessive gene located on linkage group 10 (Kobayashi et al., 2014), a gene on chromosome 7 influences the preference for *Bph1* plants, and two major QTLs on chromosomes 5 and 14 influence the insect’s growth rates on resistant rice plants (Jing et al., 2014). The BSR-seq approach in this study extended these findings by identifying 24 SNPs linked with BPH virulence on six chromosomes. Most of these SNPs are located on chromosomes 3, followed by chromosomes 13, 4, 7, 8, and 14 (Supplementary Table 6). Furthermore, two chromosome regions (Chr3, 73413752-83702207 and 88874910-94058321) were identified (Figure 6). All the results suggest that the mechanism underlying BPH virulence is complex and involves multiple genes. In this analysis, we crossed a virulent female from biotype Y and an avirulent male from biotype 1 to produce an F2 population and performed a BSR-sequencing between the two extreme bulks. In the future, more BPH crosses and repeated BSR-sequencing analysis should be carried out to confirm and fine map the identified loci. Loci harboring the SNPs and candidate genes are expected to greatly facilitate determination of mechanisms involved in BPH virulence.

## Conclusion

We identified 751 DEGs between the virulent and avirulent BPHs and mapped 24 SNPs linked to BPH virulence to rice plants with *Bph15*-mediated resistance by BSR-seq analysis. Functional analysis of 418 DEGs highly expressed in the avirulent insects showed that BPH upregulates carbohydrate, amino acid, and nucleotide metabolism, endocrine system, and signal transduction pathways in response to feeding on these plants. Lipid metabolism and digestive system pathways may play roles for BPH adaption to resistant rice. We expect the identification of relevant genetic loci and genes presented here to facilitate further research on BPH virulence mechanisms and responses to resistant plants.

## Data Availability Statement

The datasets presented in this study can be found in online repositories. The names of the repository/repositories and accession number(s) can be found below: https://www.ncbi.nlm.nih.gov/, PRJNA792102.

## Author Contributions

WG and GH designed the research, analyzed data, and wrote the manuscript. WG performed most of the experiments. JS, MG, JG, DW, QZ, JW, RC, BD, LZ, and GH performed some of the experiments. All authors read and approved the manuscript.

## Conflict of Interest

The authors declare that the research was conducted in the absence of any commercial or financial relationships that could be construed as a potential conflict of interest.

## Publisher’s Note

All claims expressed in this article are solely those of the authors and do not necessarily represent those of their affiliated organizations, or those of the publisher, the editors and the reviewers. Any product that may be evaluated in this article, or claim that may be made by its manufacturer, is not guaranteed or endorsed by the publisher.

## Footnotes

1
http://samtools.sourceforge.net/mpileup.shtml

2
https://daehwankimlab.github.io/hisat2/
