# Supplementary material for: Bulked Segregant RNA Sequencing Revealed Difference Between Virulent and Avirulent Brown Planthoppers
Source: Front Plant Sci. 2022 Apr 14;13:843227. doi: 10.3389/fpls.2022.843227 (PMC9047503; doi:10.3389/fpls.2022.843227)
Supplement: Supplementary file 1 [file Data_Sheet_1.zip › Supplementary Figures.pdf]

Supplementary Material

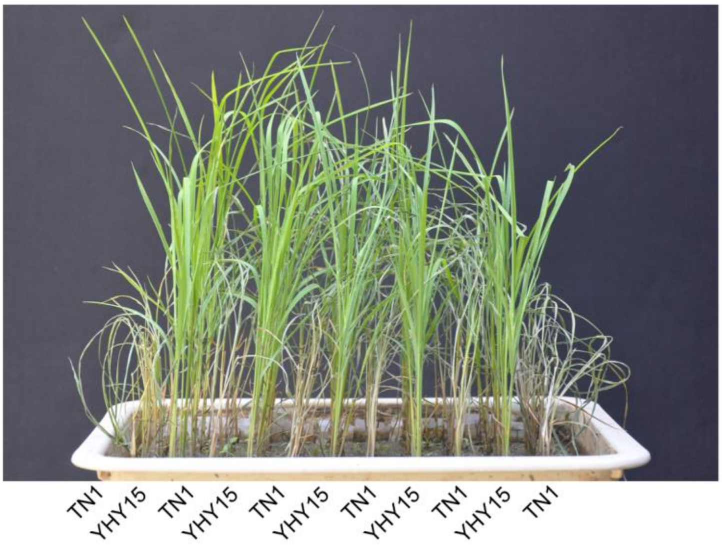

Figure S1 Verification of BPH resistance of YHY15 plants carrying the *Bph15* resistance gene. YHY15 rice plants showed a high level of resistance to BPH insects at the seedling stage. Three independent experiments were performed.

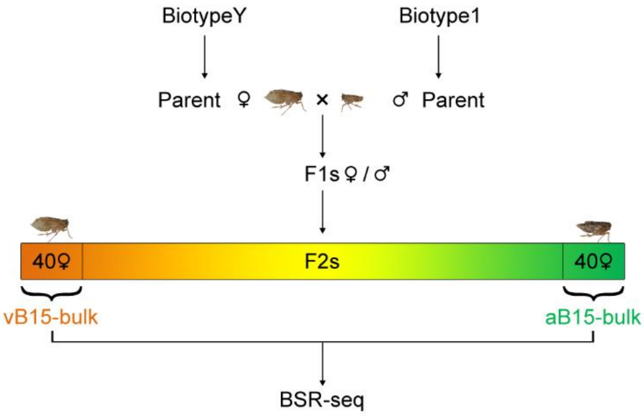

Figure S2 Schematic of sample preparation for Bulked segregant RNA-seq analysis. BPH biotype Y and biotype 1 were used for constructing the bulks. A virulent female selected from biotype Y was mated with an avirulent male from biotype 1. The F1 insects were intercrossed to breed F2 population. Forty virulent F2 females (with weight gain from 1.89 to 2.8 mg) and 40 avirulent F2 females (weight gain from -0.4 to -1.04 mg) were selected to construct the vB15-bulk and aB15-bulk, respectively.

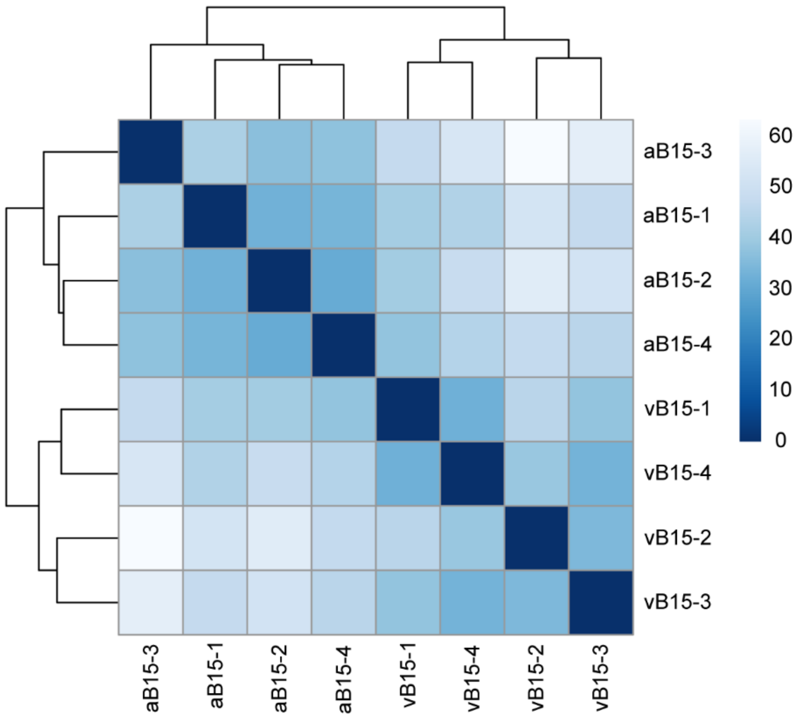

Figure S3 Validation of the inter-sample correlation. Correlation analysis between the RNA samples showed that these replicates correlated well.

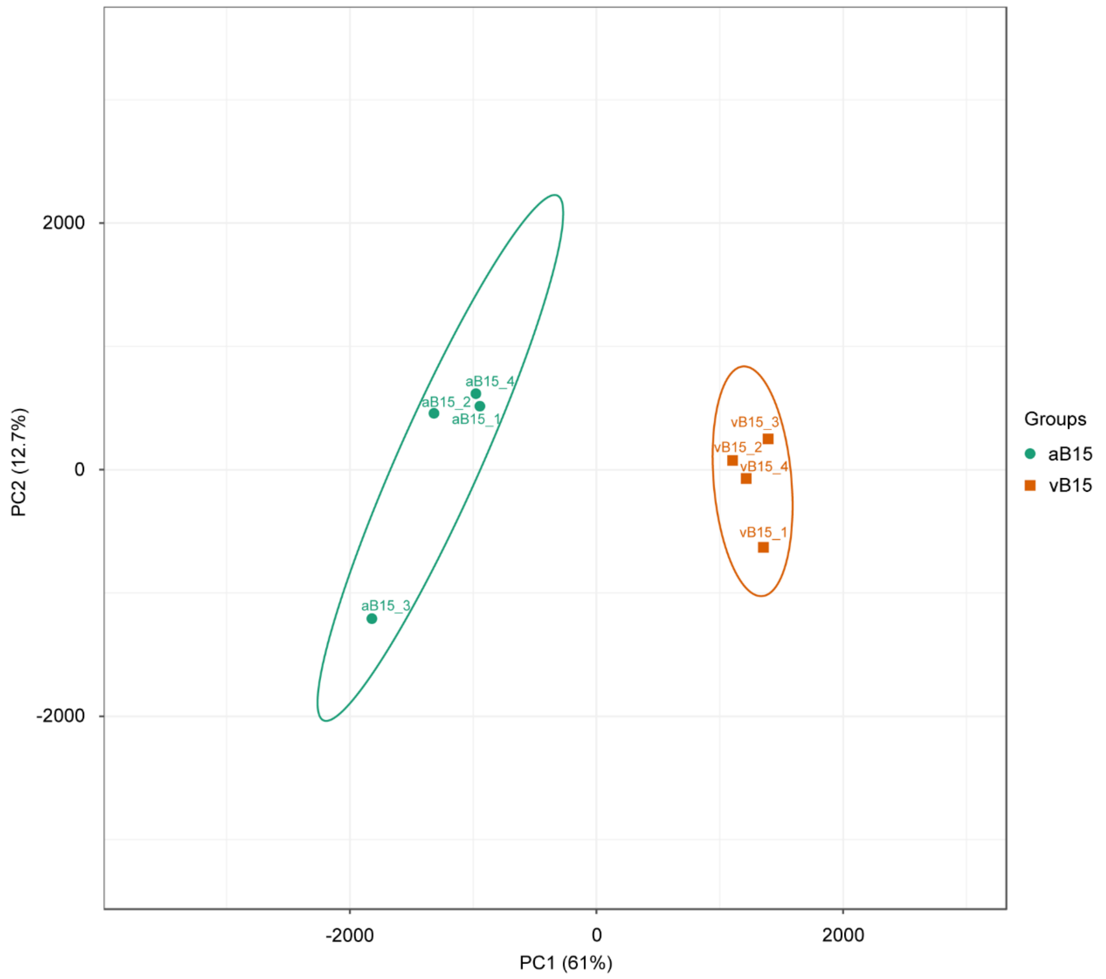

Figure S4 Principal components analysis (PCA) of the RNA-seq data set of 8 samples. The first two principal components (PC1 and 2) explained 73.7% of the total variation detected in the RNA-seq data.

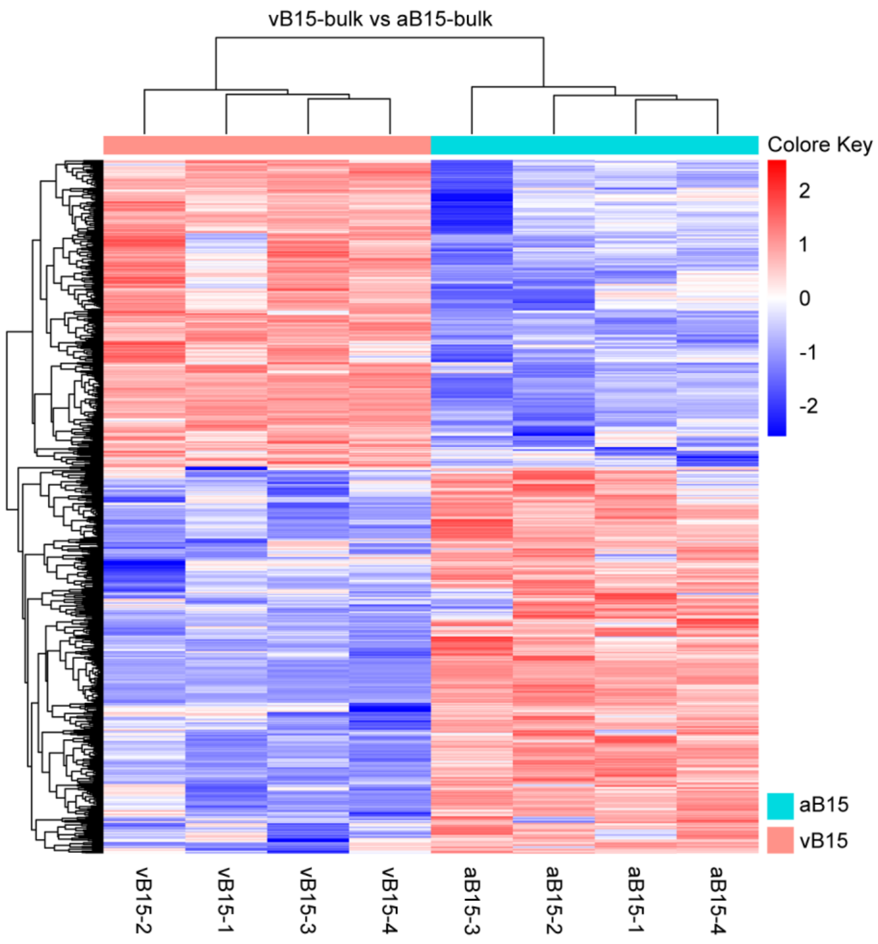

Figure S5 Expression-level clustering of the DEGs. Comparison of vB15-bulk and aB15-bulk gene expression profiles identified 751 DEGs with fold change (FC) ⩾ 2 and $P < 0.05$.

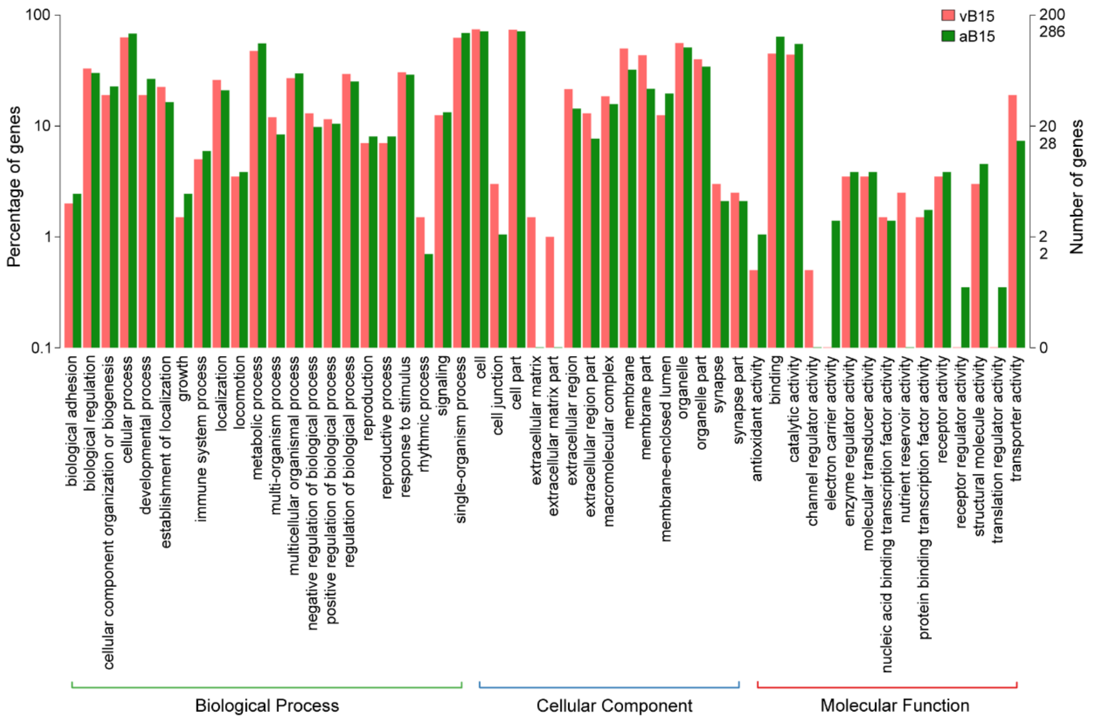

Figure S6 Histogram of Gene Ontology (GO) classes of the DEGs. The DEGs preferentially expressed in aB15-bulk and vB15-bulk were assigned to 48 and 49 functional groups, respectively. The level 2 GO terms are shown.

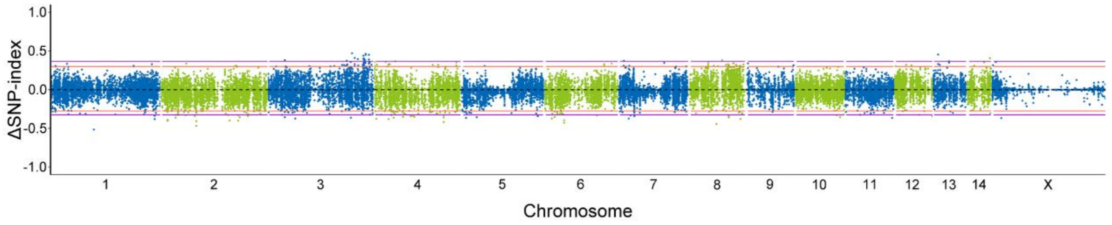

Figure S7 ΔSNP-index graph of BSR-seq. The ΔSNP-index was obtained by subtracting the aB15 bulk SNP index frome the vB15 bulk SNP index. The X-axis represents the position of the BPH chromosomes, and the Y-axis represents the ΔSNP-index. The SNP index was calculated in 3-Mb window size and 1-Mb slide size. The ΔSNP-index plot was constructed with statistical confidence intervals under the null hypothesis. The orange line indicates the threshold ($P < 0.05$), and the purple line indicates the threshold ($P < 0.01$).
